# A general approach to risk modeling using partial surrogate markers with application to perioperative acute kidney injury

**DOI:** 10.1186/s41512-017-0022-1

**Published:** 2017-12-28

**Authors:** Derek K. Smith, Loren E. Smith, Frederic T. Billings, Jeffrey D. Blume

**Affiliations:** 1 Department of Biostatistics, Vanderbilt University Medical Center, 2525 West End Ave, Suite 11000, Nashville, TN 37212 USA; 2 Department of Anesthesiology, Vanderbilt University Medical Center, Nashville, TN USA

**Keywords:** Surrogate markers, Mixture models, Acute kidney injury, Serum creatinine

## Abstract

**Background:**

Surrogate outcomes are often utilized when disease outcomes are difficult to directly measure. When a biological threshold effect exists, surrogate outcomes may only represent disease in specific subpopulations. We refer to these outcomes as “partial surrogate outcomes.” We hypothesized that risk models of partial surrogate outcomes would perform poorly if they fail to account for this population heterogeneity. We developed criteria for predictive model development using partial surrogate outcomes and demonstrate their importance in model selection and evaluation within the clinical example of serum creatinine, a partial surrogate outcome for acute kidney injury.

**Methods:**

Data from 4737 patients who underwent cardiac surgery at a major academic center were obtained. Linear and mixture models were fit on maximum 2-day serum creatinine change as a surrogate for estimated glomerular filtration rate at 90 days after surgery (eGFR90), adjusted for known AKI risk factors. The AUC for eGFR90 decline and Spearman’s rho were calculated to compare model discrimination between the linear model and a single component of the mixture model deemed to represent the informative subpopulation. Simulation studies based on the clinical data were conducted to further demonstrate the consistency and limitations of the procedure.

**Results:**

The mixture model was highly favored over the linear model with BICs of 2131.3 and 5034.3, respectively. When model discrimination was evaluated with respect to the partial surrogate, the linear model displays superior performance (*p* < 0.001); however, when it was evaluated with respect to the target outcome, the mixture model approach displays superior performance (AUC difference *p* = 0.002; Spearman’s difference *p* = 0.020). Simulation studies demonstrate that the nature of the heterogeneity determines the magnitude of any advantage the mixture model.

**Conclusions:**

Partial surrogate outcomes add complexity and limitations to risk score modeling, including the potential for the usual metrics of discrimination to be misleading. Partial surrogacy can be potentially uncovered and appropriately accounted for using a mixture model approach. Serum creatinine behaved as a partial surrogate outcome consistent with two patient subpopulations, one representing patients whose injury did not exceed their renal functional reserve and a second population representing patients whose injury did exceed renal functional reserve.

**Electronic supplementary material:**

The online version of this article (10.1186/s41512-017-0022-1) contains supplementary material, which is available to authorized users.

## Background

Patient level clinical risk score development and associated decision support applications are vitally important to modern personalized medicine. For many pathologies, this process is straightforward. First, the disease process of interest is defined and the data about relevant covariates are collected. This information is used to develop a statistical model that meets desired performance measures. When the disease process is difficult to directly measure, however, surrogate measurements are often used which prevent the use of simple risk score modeling methodology.

In 1989, Prentice defined necessary surrogate outcome criteria to ensure valid hypothesis testing [1]. Further work on surrogate outcome criteria has focused on the preservation of type I error rates for inference [2]. Surrogate outcome criteria for the development of risk scores, however, remain undefined. These criteria will be developed in Section 2 of this work.

After delineating criteria for the use of surrogate markers in risk score development, Section 3 will examine the increased modeling complexity associated with partial surrogacy situations. Partial surrogates are a class of markers that behave differently in different patient subpopulations. In one subpopulation, partial surrogates may display a high association with the outcome of interest, while in others, they may display no association. In this study, we examine how serum creatinine change for the assessment of acute kidney injury behaves in this manner. However, there are many other commonly used clinical markers that scientific considerations make suspect for partial surrogacy. For example, liver enzymes are often used to measure acute liver injury in the same way serum creatinine change is used to measure acute kidney injury. Unlike serum creatinine, liver enzymes are a direct biomarker for liver damage entering the blood stream as a direct result of liver cell death. Although it might seem like that would preclude partial surrogate behavior, there are subpopulations where liver enzymes behave differently than in health people. When used as a surrogate for acute liver injury in patients with cirrhosis, the utility of the marker is greatly reduced because in this subpopulation liver damage often resulting in little to no enzyme production. Fitting a predictive model meant to quantify acute liver injury in a population that contains both healthy patients and patients with cirrhosis would likely demonstrate the same type of partial surrogate behavior noted in the AKI example, but instead of arising from a threshold which the injury must overcome to be detectable, it would likely demonstrate a ceiling effect which chronic liver disease patients may have exceeded. Some other examples of markers for which scientific considerations might imply partial surrogacy include alveolar bone loss for the assessment of periodontal disease severity and ST elevations for the assessment of myocardial infarct in a population containing patients with left bundle branch block.

The heterogeneity displayed by partial surrogates between relevant subpopulations influences their ability to satisfy Prentice’s criteria for hypothesis testing. We demonstrate that partial surrogate outcomes also complicate our proposed surrogate criteria for risk score prediction. Additionally, evaluating risk scores using a partial surrogate is complicated by the observation that the model which provides optimum discrimination for the surrogate outcome does not necessarily discriminate the target outcome. The implications of this observation for model selection and evaluation of likely clinical benefit will be described. Finally, Section 4 of this manuscript will explore analytical challenges introduced by partial surrogacy theoretically and computationally.

In the course of this work, an analysis of perioperative acute kidney injury (AKI) will be performed to emphasize the clinical importance of our surrogate criteria for risk score modeling and to demonstrate the limitations and special considerations associated with partial surrogates in an applied analysis context.

### Section 2: surrogate outcomes in risk score models

Clinical risk scores are commonly assessed in two ways. The first way in which they are assessed is by model discrimination, the degree to which a risk score is ordered similarly to the disease marker of interest. Second, they are assessed by model calibration, a comparison of the magnitude of the risk score and the magnitude of the disease marker of interest. Risk scores that are well calibrated are simpler to implement and are traditionally considered ideal due to the observation that good calibration generally implies good discrimination. Unfortunately, there is no reason to expect that a risk model built on a surrogate measure will be well calibrated as it is designed to predict the surrogate and not the target outcome. For this reason, the risk scores developed here will be evaluated on measures of pure discrimination (area under the receiver operating curve for binary measures and Spearman’s rho for continuous ones) as opposed to more traditional measures of predictive performance such as mean square error, which are sensitive to calibration.

Suppose that we are interested in developing a risk score, *R*, for a true clinical outcome, *T*, where *R* is any one-dimensional summary of a patient’s data that is intended to help quantify a patient’s disease state disposition. Next, suppose that we are unable to measure *T* itself in the timeframe necessary to develop a useful decision support tool. Finally, suppose a surrogate outcome, *S*, is readily measurable and related to *T* either as a mediator or a consequence which presents more readily.

While developing *R*, our goal will be to obtain a one-dimensional summary of the data that discriminates well and maintains some interpretability of the model coefficients as these are often used to generate hypotheses about potential mechanisms. A score, *R,* should provide higher scores for higher risk or more severely diseased patients uniformly over the entire range of plausible scores. We will refer to this last property as being clinically useful. Ideally, clinical utility should be consistent over the entire range of potential risk scores. Otherwise, *R*’s discriminatory ability might look favorable when examined over the entire population, despite *R* preforming poorly for a particular subset of patients. This could result in a net-benefit to the population at the expense of a particular group of individuals, raising questions about the ethical implementation of *R* for generalized patient care.

What criteria of *S* which make the resulting risk score more likely to be clinically useful? Although Prentice’s criteria have been criticized for being overly stringent for practical application, we will use them here to aid in the development of a less restrictive set of criteria for the evaluation of surrogates for risk stratification. Following the pattern of Prentice’s first and second criteria for valid hypothesis testing [1], surrogate endpoints must display a relationship between the suspected risk factors to be included in the model, *Z,* and both the surrogate and target outcomes, *S* and *T*, respectively. Stated more formally, the conditional distributions of *S* and *T* on *Z* must not be equal to the marginal distributions over *Z*. For clarity, Prentice’s criteria will be labeled with *P*, and the prediction criteria will be labeled with an *R*.

- *P*_1_. The proposed risk factor is related to the surrogate *f*(*S*| *Z*) ≠ *f*(*S*).
- *P*_2_. The proposed risk factor is related to the target outcome *f*(*T*| *Z*) ≠ *f*(*T*).

The necessity of these two criteria, which when applied to risk score procedures will be referred to as *R*_1_ and *R*_2_, respectively, is fairly evident. A failure of criterion *R*_1_ suggests that the covariates included in the predictive model contain no information about the distribution of the surrogate. As such, models built on the surrogate would display little variation in the risk score *R|Z* and any variation observed would be random. A failure of criterion *R*_2_ suggests that the covariates are not related in any way to the distribution of the target outcome, and although *R|Z* may display a rich variation, it would be expected that *f*(*T*| *R*, *Z*) = *f*(*T*).

For risk score development, a third, less restrictive relationship between variables is necessary in order to obtain good discrimination and produce a clinically useful model. It is desirable that the distribution of *R* ∣ *T* be changing to favor more extreme values as *T* increases. Therefore, for some *T*_1_ < *T*_2_ corresponding to risk scores *R*_*1*_|*T*_*1*_ and *R*_*2*_|*T*_*2*_, we have that

- *R*_3_. *P*(*R*_1_ < *R*_2_ | *T*_1_, *T*_2_) > 0.5.

The *R*_3_ criterion promotes variation in *R* over different values of *T*. This ensures that, on average, the risk score is producing more extreme values when *T* is more extreme.

Ideally, the probability described in *R*_3_ would be large. This occurs when the locational shift in the distribution of *R*|*T* as *T* changes is large relative to its variance. Although not a strict requirement, having a risk score that is precise will naturally enhance its value.

In summary, our criteria for the development of a surrogate outcome-based risk score are:

- *R*_1_. The proposed risk factor is related to the surrogate *f*(*S*| *Z*) ≠ *f*(*S*).
- *R*_2_. The proposed risk factor is related to the target outcome *f*(*T*| *Z*) ≠ *f*(*T*)
- *R*_3_. The distribution of the risk score conditional on *T* needs to be shifting toward more extreme values amongst those at highest risk for disease

$$ P\left({R}_1\left\langle {R}_2\right|{T}_1,{T}_2\right)>0.5,{T}_1<{T}_2, $$

with Prentice’s 3rd and 4th criteria and the magnitude of the variance of *R* ∣ *T* relative to its distributional shift being unnecessary but playing roles in determining the value of the resultant score.

These criteria encompass a surrogate outcome’s minimum requirements to produce a valid risk score. In the next section, we will begin to examine partial surrogates, and how the failure of some of these criteria in patient subpopulations can negatively impact risk score performance.

### Section 3: theoretical considerations regarding partial surrogacy and risk score modeling

In the ideal situation, *R*_1_–*R*_3_ would hold in every subpopulation on which a risk score model is to be trained. In other words, it is beneficial if the phenotype defined by the relationship between *Z, S,* and *T* is homogenous throughout a population, **P**. However, if there are subpopulations demonstrating differing phenotypes, extra care is required to maximize the benefit of risk score models and provide valid estimation procedures. When these heterogeneous subpopulations exist, we will redefine *S* to be a “partial surrogate.”

As an example, suppose you have collected data from **P** which is composed of two subpopulations **V** and **I**, defined by a latent indicator variable, *l*. In subpopulation **V**, *R*_1_–*R*_3_ hold, suggesting subpopulation **V** might produce a valuable risk score model. In subpopulation **I**, however, only *R*_2_ holds. This suggests that in subpopulation **I**, *S* is not meaningfully related to *Z* or *T* and is therefore unlikely to result in a profitable risk score in this subpopulation.

The ideal method for risk score development when faced with a partial surrogate is not immediately apparent. One method is to use traditional modeling strategies in the full training dataset. In cases where the full dataset satisfies *R*_1_–*R*_3_, this approach is likely to result in valuable models. If *l* was known, an analyst might reasonably decide to use only the data from subpopulation **V** for model development and then generalize the model to the entire population as appropriate. This second method relies on the relationship between *T* and *Z* being homogeneous over **P**. Homogeneity will occur if the subpopulations were defined completely at random. Alternatively, in cases where *l* is unknown, a latent variable mixture model can be used to produce a similar result. For the duration of this manuscript, *l* is assumed to be latent.

Given these two approaches, the analyst is forced to choose between the full-data approach and the mixture model approach. For inference and estimation, the choice is clear. Since failing to account for the partial nature of the surrogate will result in a violation of Prentice’s 4th criterion, the mixture model is preferable. For example, consider a very simple partial surrogate where

$$ T=S\left|V+{\varepsilon}_{T\mid S}\ \mathrm{and}\ S\right|I={\varepsilon}_{S\mid I},{\varepsilon}_i\sim N\left(0,{\sigma}_i\right) $$

and also

$$ T\mid Z={\beta}_{T\mid Z}+{\beta}_1Z+{\varepsilon}_{T\mid Z}. $$

In this situation, the surrogate is equal to truth plus error when a patient belongs to subpopulation **V**, but it is a random deviate when the patient is from subpopulation **I**. The relationship between *T* and *Z* is consistent across the entire population. Thus, we have

$$ E\left[T\left|S\right.\right]=P(V)\ S+P(I)\ E\left[T\right]=P(V)S+\left(1-P(V)\right)E\left[T\right] $$

and also that

$$ E\left[T|S,Z\right]=P(V)S+P(I)E\left[T|Z\right]=P(V)S+\left(1-P(V)\right)\left({\beta}_{T\mid Z}+{\beta}_1Z\right). $$

*P*_4_ requires that the distribution of *T*|*S* be the same as the distribution of *T*|*S, Z*, but even this simple partial surrogate violates that criterion as evidenced by the differing expectations.

However, for risk score modeling, the decision is less clear. Using the full dataset and not accounting for the partial nature of the surrogate generally results in risk scores with lower variance due to higher effective sample size but higher bias due to the inclusion of training data from population **I**. The mixture model approach generally boasts reduced bias by correctly accounting for heterogeneous subpopulations but suffers higher variance due to diminished training set sample size. There are several aspects unique to a given partial surrogate situation that should affect the analyst’s decision regarding these modeling strategies.

When making the decision between using a traditional model or a mixture model, the first consideration is whether the added complexity of the mixture model approach is likely to be beneficial. The mixture model’s primary purpose is to estimate covariate/outcome relationships in the subpopulations separately. In order for this to practically improve the risk score’s discrimination, it needs to result in a different rank ordering of subjects compared to the traditional approach. This is likely to occur whenever the phenotype expressed in subpopulation **I** is substantively different than that in subpopulation **V** in terms of the relative magnitude of the associations between the covariates and outcome. This distinctness of subpopulation phenotypes simultaneously allows the expectation maximization (EM) algorithm used for model fitting to achieve adequate subpopulation separation while achieving a more appropriate ordering of predictions with respect to *T*.

In order to apply the mixture modeling approach to a clinical problem, it is necessary to decide how many components the model should have. In the case of partial surrogates, this is the number of subpopulations that are present. In many cases, the number of subpopulations may be strongly suspected based on clinical considerations, but in cases where the number is less certain, there is a large literature that describes various methods for identifying the proper number of components and the consequences of selecting the wrong number [3–6].

The EM algorithm involves beginning with a prior probability of group assignment, fitting a model that is weighted by the prior probability to assess the likelihood of subpopulation membership, and calculating a posterior probability of subpopulation membership based on the prior and the likelihood. This is repeated until convergence is achieved with the posterior probabilities being used to generate the prior probabilities for the next iteration [7]. Substantial separation between subgroup phenotypes results in the EM algorithm calculating final posterior probabilities of subpopulation membership that are close to zero and one, suggesting there is good evidence in the data to direct each patient’s subpopulation assignment. When the subpopulations cannot be effectively separated, mixture model variance will be magnified, detracting from its utility and favoring the traditional modeling approach.

A second consideration affecting the development of partial surrogate-based risk scores is how generalizable a subpopulation model based on **V** will be to the entire population **P**. If separation into subpopulations **I** and **V** is completely random, then any result obtained from subpopulation **V** should be fully generalizable. If subpopulations **I** and **V** are generated by a non-random process, however, neither modeling technique considered above is guaranteed to result in a clinically beneficial risk score, and additional external verification would be necessary to allow generalization.

The last major consideration that influences whether the mixture model approach is viable for risk score development with partial surrogates is the mixing proportion of the population. It is necessary to estimate what proportion of observations is from **V** versus the proportion from **I**. If the training data are composed almost entirely of data from **V**, the mixture model adds little benefit over the traditional model which ignores subpopulations. In contrast, if the data are almost entirely from **I**, there may not be enough information in the data to accurately fit a model for subpopulation **V**, which embodies the clinically relevant covariate/outcome relationship. In both of the situations described here, partial surrogate-based risk score models are unlikely to provide a benefit over the traditional modeling approach because the available dataset does not contain enough information regarding the true relationship between covariates and the outcome of interest.

In summary, there is no universal solution to measuring pathology with partial surrogate outcomes. The mixture model approach provides great benefits in some situations, but in others, the mixture model approach fails to adequately fit the data and will lead to inferior performance compared to a more traditional, non-mixture approach.

### Section 4: examination of developed risk score criteria and partial surrogate guidelines through clinical analysis and simulation studies of perioperative AKI

#### Biological background

**Fig. 1:**
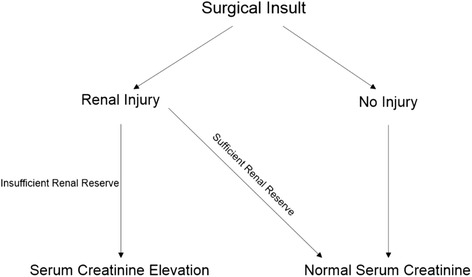
Directed acyclic graph displaying the hypothesized mechanism which would result in statistically heterogeneous subpopulations with respect to serum creatinine change

Ten to 40% of patients develop AKI following major inpatient surgical procedures [8]. Perioperative AKI has been associated with increased short and long-term mortality, increased hospital length of stay, increased risk of developing chronic kidney disease (CKD), and increased risk of developing dialysis dependence [8, 9]. Unlike other perioperative injuries, such as myocardial infarction, there is currently no direct biomarker of kidney injury or cell death that accurately and consistently reflects AKI. Current consensus guidelines for AKI diagnosis use changes in serum concentrations of creatinine to diagnose AKI [10]. Creatinine is produced by the muscle, and the kidneys excrete creatinine. Injured kidneys excrete less creatinine, and increased serum concentrations of creatinine are used to diagnose AKI. The relationship between AKI and serum creatinine is inconsistent, hindering accurate AKI diagnosis. One example of this inconsistency is the clinical situation in which renal injury does not produce an increase in serum creatinine (Fig. 1). Patients often sustain kidney damaged without associated changes in serum creatinine, referred to as subclinical AKI [11]. This situation is illustrated well by the following example. A living kidney donor frequently will experience little to no serum creatinine increase following donor nephrectomy despite removal of roughly 50% of their functional kidney mass [12]. Recent AKI biomarker studies demonstrated that subclinical AKI is also associated with an increased risk of dialysis and in-hospital mortality, suggesting it represents clinically significant levels of renal injury [13]. The accurate measurement of subclinical AKI using common clinical labs in the immediate postoperative period (creatinine) would allow physicians to predict AKI, institute additional patient monitoring, and adjust patient treatments. These benefits could reduce patient morbidity and mortality.

The dramatic example of living donor kidney donation and minimal serum creatinine change demonstrates that healthy kidneys have the capacity to temporarily increase their filtration rate in times of physiologic stress, a characteristic termed renal functional reserve [14]. Renal functional reserve, however, is difficult to predict, difficult to measure, limited, and exhaustable [14]. In the subpopulation of patients, **V**, who overcome their renal functional reserve during kidney injury serum creatinine change would be detected, and associations between relevant risk factors and serum creatinine change would be strong, assuming all other serum creatinine modifying factors remain constant. In the subpopulation of patients, **I**, who do not overcome their renal functional reserve during episodes of kidney injury, only random or nonspecific changes in serum creatinine levels would be measured, and the associations between relevant AKI risk factors and serum creatinine change would be weak. With respect to the proposed risk score criteria outlined in Section 2, this suggests that subpopulation **V** will likely come close to satisfying *P*_1_–*P*_4_ and *R*_1_–*R*_3_, allowing for simultaneous estimation of associations and risk score generation. In contrast, subpopulation **I** will likely violate *P*_1_, *P*_3_, *P*_4_, *R*_1_, and *R*_3_, resulting in poor performance of risk indices based exclusively on this subgroup, biased coefficient estimates, and improper *p* values.

If subpopulations **I** and **V** are defined based on exhaustion of renal functional reserve as we hypothesize, then it is important to recognize that the likelihood of renal functional reserve exhaustion is not random. Young, healthy patients are less likely to overcome their substantial renal reserve than older patients with underlying disease [14, 15]. Therefore, generalizing a risk score generated in subpopulation **V** to the entire population **P** requires validation of that score in the entire population. This validation can be accomplished by evaluating the partial surrogate-based clinical risk score’s discrimination of the target outcome, *T*. Although there is no gold standard marker for clinically significant kidney damage, one marker of indisputable clinical significance is the decline in kidney glomerular filtration at 90 days [16, 17]. The 90 days following surgery allows the kidneys to recover from acute injury if possible and reestablish an equilibrium serum creatinine concentration. This postoperative day 90 serum creatinine concentration is used to estimate glomerular filtration rate via the Chronic Kidney Disease Epidemiology Collaboration equation (eGFR90) [18], the primary indicator of kidney function. Indeed, current clinical guidelines recommend that patients who experience AKI should routinely have 90 day eGFR evaluation to assess recovery versus progression to permanent kidney damage [19]. Therefore, in this analysis eGFR90 will be considered the target outcome, *T*.

## Methods

### Data and models

The data used in this analysis are from 4737 patients who underwent cardiac surgery at a large academic medical center from November 2009 through June 2015. Institutional IRB approval was obtained prior to performance of all analyses. In this dataset, all patients had serum creatinine measurements in the first two post-operative days and 1268 patients had 90 ± 15 day eGFR90 measurements available. Table 1 compares the characteristics between those with and without a recorded value for eGFR90. Aside from a slight deviation in the proportion of patients with diabetes, the covariates are well balanced between the groups making a missing at random assumption plausible. However, sensitivity to this assumption will also be assessed.

**Table 1:** Patient and surgical characteristics stratified by whether the patient record contained a record of eGFR at 90 days postoperatively. Continuous variables are reported as median (IQR) and binary variables are reported as proportion (%)

	eGFR90 present	eGFR90 missing
*n*	1268	3469
Age (year)	61 [51, 69]	64 [54, 71]
BMI (kg/m^2^)	29.14 [25.02, 33.80]	28.41[25.00, 32.72]
Hemoglobin (g/dL)	12.4 [10.5, 13.9]	13.3 [11.8, 14.6]
Diabetes	567 (44.7%)	1198 (34.5%)
Total urine output (mL)	450 [275, 725]	450 [300, 700]
Total fluids given (mL)	1600 [1000, 2200]	1750 [1100,2400]
Baseline eGFR	66.69 [47.06, 85.95]	74.52 [56.62, 90.24]
Max intraoperative lactate (mg/dL)	1.9 [1.2, 3.2]	1.6 [1.0, 2.6]
Length of surgery (min)	318 [260, 404.25]	301 [253, 368]
Emergency surgery	113 (8.9)	202 (5.8)

Ten preoperative and intraoperative traits were selected a priori for inclusion in the analysis including age, body mass index, a diagnosis of diabetes, baseline kidney (glomerular) filtration rate, baseline hemoglobin concentration, volume of intraoperative urine output, volume of intraoperative intravenous fluid administered, maximum measured intraoperative plasma lactate level, length of surgery, and an indicator for emergent surgery. These variables were chosen as well-established predictors of AKI and therefore were considered likely to be valuable predictors of serum creatinine change from baseline [20–24].

For the purposes of model comparisons, a linear model and a two-component mixture of linear models were fit. The residual error of the two mixture components was not constrained. The linear model risk score is the model’s prediction. For the mixture model, the risk score is the prediction from the single component of the mixture that is post hoc identified to be associated with subpopulation **V**. Each model was evaluated based on the following metrics: the AUC for a target outcome greater than 20 mL/min/1.73 m^2^ and the Spearman’s correlation. The first metric is a common method of risk score implementation and is based on the presumption that a change of 20 mL/min/1.73 m^2^ in eGFR90 is clinically meaningful. An absolute change in eGFR90 was chosen over a relative change because of the many statistical issues that can arise from the inclusion of ratios of random variables such as improper error distributions and spurious associations [25]. For comparison, we have included the ROC curves that would result for the models predicting whether 2-day postoperative serum creatinine change exceeded 0.3 mg/dL, a relatively sensitive cutoff for AKI suggested by the Acute Kidney Injury Network and the Kidney Disease Improving Global Outcomes guidelines [19, 26]. The second metric measures discrimination without requiring an arbitrary cutoff.

In order to assess the sensitivity of this analysis to the missingness of eGFR90 and the choice of cutoff in eGFR90 for the ROC analysis, an additional analysis was performed. A logistic propensity score model was fit to whether eGFR90 was present in the patient record. The resulting propensity score was used as a weight, and the AUC was recalculated. This process was repeated at each potential cutoff in eGFR90 from 5 to 25 mL/min/1.73 m^2^ resulting in a propensity score adjusted, eGFR90-dependent ROC curve.

## Results

The mixture model resulted in moderately well-differentiated clusters and a relative entropy equal to 0.607. Seven hundred twenty-eight patients were modally assigned to the **V** subpopulation, and 4009 patients were assigned to the **I** subpopulation. The linear model found all the factors to be significantly associated with eGFR90 change except for a history of diabetes and emergency surgery, which were marginally significant (*p* = 0.085 and *p* = 0.063, respectively). The mixture component found all the risk factors to be significantly associated with eGFR change with the exception of emergency surgery (*p* = 0.072). However, the magnitude of the coefficients for the linear model were attenuated by an average of 42.8% (range = [19.2%, 68.1%]), which is consistent with subpopulation **I**’s phenotype being attenuated relative to subpopulation **V**. The model coefficients are given in Table 2. In addition, the mixture model represented a substantial improvement in fit over the linear model with BICs of 2131.3 and 5034.3, respectively.

**Table 2:** Coefficients resulting from the application of the mixture model to the perioperative AKI dataset. The column on the right represents the coefficients from subgroup V and are noticeably larger in absolute magnitude than those on the left

	Subpopulation I	Subpopulation V
BMI	0.047 (0.038, 0.057)	0.25 (0.206, 0.295)
Total urine output	− 0.041 (− 0.052, − 0.03)	− 0.147 (− 0.193, − 0.100)
Total fluids given	− 0.015 (− 0.025, − 0.004)	− 0.134 (− 0.179, − 0.09)
Age	0.063 (0.051, 0.074)	0.213 (0.168, 0.258)
Baseline eGFR	0.110 (0.097, 0.122)	0.388 (0.344, 0.433)
Hemoglobin	− 0.063 (− 0.074, − 0.053)	− 0.253 (− 0.297, − 0.209)
Max intraoperative lactate	0.060 (0.048, 0.073)	0.206 (0.161, 0.250)
Diabetes	− 0.002 (− 0.013, 0.008)	− 0.109 (− 0.152, − 0.066)
Length of surgery	0.072 (0.058, 0.085)	0.193 (0.144, 0.241)
Emergency surgery	0 (− 0.011, 0.011)	0.05 (0.012, 0.087)

**Fig. 2:**
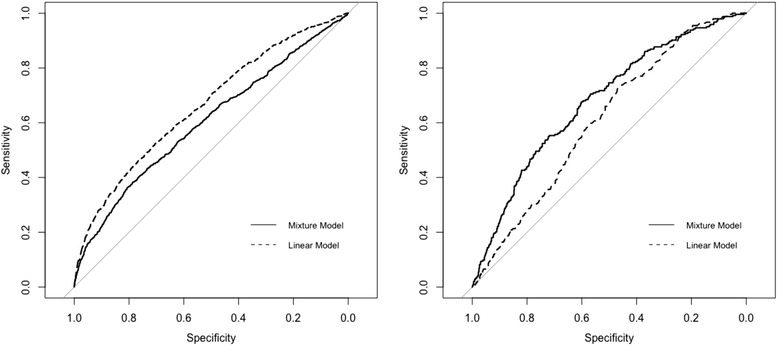
Receiver operating characteristic curves for the linear (dashed) and mixture (solid) models predicting maximum 2-day creatinine change (left) vs. eGFR90 (right)

The area under the ROC curve was calculated for each of the candidate risk scores and for the gold standard of the observed serum creatinine change for the prediction of an eGFR90 decline greater than 20 mL/min/1.73 m^2^. The observed serum creatinine change had the worst estimated AUC of 0.608 (0.572, 0.645), although not significantly worse than that of the linear model 0.633 (0.595, 0.672), *p* = 0.262. The mixture model component yielded the best AUC of 0.678 (0.641, 0.715), which was a significant improvement over both the observed creatinine change and the linear model, *p* = 0.002 and *p* < 0.001, respectively. In addition, the ROCs were calculated for each candidate risk score for the prediction of a serum creatinine increase greater than 0.3 mg/dL. The result was an AUC of 0.602 (0.582, 0.623) for the mixture and 0.663 (0.644, 0.682) for the linear model, *p* < 0.001. The ROCs for both endpoints are given in Fig. 2.

The improvement due to using the mixture component’s prediction as a risk score for eGFR90 is further demonstrated by looking at Spearman’s rank correlation. The correlation between the observed serum creatinine change, and the observed eGFR90 change was 0.231 (95% CI 0.204, 0.258). For the linear model, the correlation was 0.223 (0.196, 0.250). For the mixture component, the correlation was 0.305 (0.280, 0.331). These values were compared via a permutation test showing a significant improvement by the mixture model over the observed value and the linear model’s prediction, *p* = 0.035 and *p* = 0.020, respectively. The low values of these correlations are due to the fact that the majority of surgical patients sustain no kidney injury; and thus, any change in their eGFR is truly random, i.e., only a small portion of the population’s eGFR changes are ordered by something other than random chance, so despite the low correlation the improvement provided by utilizing the partial surrogate is substantial.

**Fig. 3:**
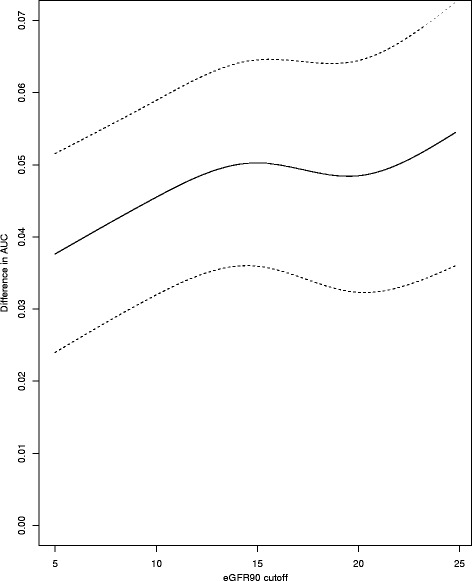
Difference in the propensity score adjusted AUC over eGFR90 cutoffs ranging from 5 to 25 mL/min/1.73 m^2^

The sensitivity analysis was performed as detailed in the methods section resulting in the propensity score adjusted, eGFR90 dependent ROC curves for each model. The difference in AUCs is given in Fig. 3. The AUC for the mixture approach is consistently, significantly higher over the entire range of potential cutoffs after accounting for the propensity to be missing.

This analysis demonstrates a major issue in the development of risk scores using partial surrogate outcomes. If the partial surrogate nature of serum creatinine change had gone unrecognized in this analysis, the analyst would likely look to how well various models discriminate with respect to serum creatinine change as a preferred method for both model selection and characterization. The ROC analysis demonstrates that the analyst would then conclude that the linear model was clearly superior to the mixture model because its risk score is ordered more similarly to the surrogate measure. However, the ROC of the target outcome, eGFR90, shows the true relationship is reversed and that the mixture model produced superior ordering. It is critical to identify partial surrogates and account for them appropriately since there would be no indication of this flaw in analysis if model performance was judged solely on its ability to predict the surrogate outcome, postoperative serum creatinine elevation.

### Simulation studies

The results of the clinical example presented above were used to generate simulation studies meant to further illustrate the potential benefits and limitations of using the mixture modeling approach in the presence of a partial surrogate. In the above example, a two-component mixture model was fit to the data resulting in two fitted models, ${\widehat{f}}_V$ and ${\widehat{f}}_I$, with corresponding parameters $\left({\widehat{\beta}}_V,{\widehat{\sigma}}_V^2\right)$ and $\left({\widehat{\beta}}_I,{\widehat{\sigma}}_I^2\right)$. Each data point represents a draw from one of these two models with a certain probability.

In each of the simulations that follow, each of the patients in the cohort will be assigned to subpopulation **V** or **I** by a random draw governed by their individual posterior probability of group membership derived from the fitted mixture model. New outcomes were then generated according to which subgroup the patient was assigned to. In both simulations, a new value of *S* and *T* are generated for patients assigned to subgroup **V** via

$$ {\displaystyle \begin{array}{l}\left.T\right|Z\sim N\left(Z{\widehat{\beta}}_V,{\widehat{\sigma}}_V\right)\\ {}\left.S\right|Z,V\sim N\left(Z{\widehat{\beta}}_V,{\widehat{\sigma}}_V\right).\end{array}} $$

That is *T* and *S* are generated from the same model with normal errors. After being drawn, the values of *T* were normalized to have a means of 0 and standard deviation of 50 in order to make them more consistent with eGFR90 changes. In subpopulation **I**, *T* is generated in the same way. The difference is in how *S* is generated in this subpopulation. In the first simulation, *S* will be generated via an alternative linear model in which the covariates are still related to *S* but not in the same way as they are under ${\widehat{f}}_V$. Each repetition of the simulation used a different model with the coefficients being drawn independently from

$$ {\beta}_{R,i}\sim N\left(0,.176\right),i=1,..,10. $$

The standard deviation for this distribution is ¼ of the range of the observed coefficients with the intention that the sampled coefficients would be of similar magnitude to those observed in the clinical example. The outcome for these patients was then sampled from

$$ S\mid Z,I\sim N\left(Z\left[{\beta}_{I,1},{\beta}_{R,1},\dots, {\beta}_{R,10}\right],{\widehat{\sigma}}_I\right). $$

The second data-based simulation is conducted similarly with one exception. In this simulation, *S* is drawn from

$$ S\mid Z,I\sim N\left(0,{\widehat{\sigma}}_I\right). $$

**Fig. 4:**
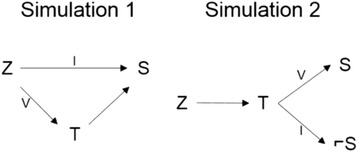
Directed acyclic graphs outlining the scenarios mimicked in the two simulation studies

This simulation represents the scenario where the covariates of interest have little to no relationship with the surrogate within subpopulation **I** but are related within subpopulation **V**. The directed acyclic graphs that describe these two scenarios are given in Fig. 4.

Having generated new outcome variables *T* and *S* for both subpopulations, a linear and two-component mixture are then fit to the simulated data. The models are compared as in the clinical example, a cutoff-based measure of discrimination (AUC), a cutoff-free measure of discrimination (Spearman’s rank correlation), and relative mean-square error (MSE) reduction in the estimation of the model coefficients. The results of these simulations are summarized in Table 3.

**Table 3:** Results of the two simulation studies given as mean (0.05 quantile, 0.95 quantile)

		AUC	Spearman’s rho	MSE
Simulation 1	Linear model	0.737 [0.518, 0.923]	0.152 [−0.091, 0.349]	0.048 [0.024, 0.077]
	Mixture model	0.950 [0.908, 0.982]	0.501 [0.471, 0.526]	0.004 [0.002, 0.008]
Simulation 2	Linear model	0.928 [0.873, 0.972]	0.394 [0.333, 0.451]	0.031 [0.028, 0.034]
	Mixture model	0.949 [0.906, 0.982]	0.492 [0.454, 0.524]	0.005 [0.002, 0.009]

In the first simulation, where the outcome for those assigned to subpopulation **I** were generated from a model that was distinct from ${\widehat{f}}_V$ but not null, the results favored application of the mixture model in each case. For the linear model, the AUC for discriminating whether *T* was larger than 20 spanned almost the entire range of the statistic [0.05, 0.95] quantiles = [0.518, 0.923]; the results for the mixture model was more consistent [0.05, 0.95] quantiles = [0.908, 0.982]. The mean difference in AUC was 0.213, [0.05, 0.95] quantiles = [0.034, 0.433]. The Spearman’s correlation displayed a similar result with the linear model yielding a wide range of values [0.05, 0.95] quantiles = [− 0.091, 0.349], whereas the mixture model produced more consistent results [0.05, 0.95] quantiles = [0.471, 0.526]. The mean difference in rank correlation was 0.349, [0.05, 0.95] quantiles = [0.146, 0.603]. Lastly, the MSE of the estimated model coefficients was substantially improved with the linear model estimating coefficients with an order of magnitude larger error [0.05, 0.95] quantiles = [0.024, 0.077] compared to the mixture approach [0.05, 0.95] quantiles = [0.002, 0.008]. This represents a relative reduction in the MSE of the coefficient estimates of 89.1%.

In the second simulation, in which the covariates are not related to the surrogate marker in patients assigned to subpopulation **I**, resulted in much more modest improvements in discrimination. In this situation, the linear model produced reasonably large AUCs consistently [0.05, 0.95] quantiles = [0.873, 0.972] although the AUCs from the mixture approach were slightly larger [0.05, 0.95] quantiles = [0.906, 0.982]. The average difference between the AUCs was 0.021, [0.05, 0.95] quantiles = [0.002, 0.044]. The linear also improved its performance with respect to Spearman’s rank correlation [0.05, 0.95] quantiles = [0.333, 0.451] compared to that of the mixture model [0.05, 0.95] quantiles = [0.454, 0.524]. With respect to the MSE of the estimated coefficients, the linear model still displayed serious bias [0.05, 0.95] quantiles = [0.028, 0.034] as compared to the mixture approach, [0.05, 0.95] quantiles = [0.002, 0.009]. This represents an 83% relative reduction in MSE.

In the first example, the surrogate is generated in subpopulation **I** in a way that by random chance can be similar to or very different from ${\widehat{f}}_V$. When the generating model is similar to ${\widehat{f}}_V$, the linear model, which pools the subpopulations together, can perform well in terms of discrimination as evident by its ability to generate high AUCs and rank correlations. However, when the generating model for subpopulation **I** is very different from ${\widehat{f}}_V$, the discrimination suffers tremendously. While the random model selection is very influential in the resulting discrimination, the estimated model coefficients produced by the linear model always represent an averaging of the true coefficients in the two models resulting in higher MSE of estimation than the mixture model approach.

In the second example, the linear model performs much better in terms of discrimination. In this example, the model coefficients estimated by the linear model are biased estimates of those in ${\widehat{f}}_V$; however, they are biased toward zero. This type of consistent attenuation preserves the relative size and direction of the coefficients. The model is therefore able to order the outcomes well with discriminatory performance only modestly lower than the mixture approach. The inclusion of subpopulation **I** in this example attenuates the estimated coefficients in the linear model resulting in a bias that inflates the MSE in a similar way to what was observed in the first simulation. This does not impact its utility as a predictive model but would have implications for inference, which is beyond the scope of this analysis. *R* code for two simplified examples of this technique is provided as a supplement to this manuscript (Additional file 1).

## Discussion

Recognizing partial surrogacy of an outcome marker is critical regardless of whether the goal of an analysis is inference or prediction. In our clinical example of perioperative AKI, it was demonstrated that treating serum creatinine change as a full surrogate rather than a partial surrogate led to the erroneous conclusion that the linear model approach was much better than a mixture model at measuring kidney injury, represented by eGFR90. Prior to this work, no account has been given to the partial surrogate nature of serum creatinine change, meaning that effect estimates and predictive models based on creatinine-related endpoints are susceptible to a severe, systematic bias.

Despite being a clinically important marker of kidney function, eGFR90 has limitations. Patients who experience transient serum creatinine elevations that resolve by 90 days may still be at increased risk for adverse sequelae [27, 28]. The analysis we performed here does not fully capture these patients’ increased risk. This analysis demonstrates that the proposed technique produced a superior model for the prediction of eGFR90. Although it is plausible that similar models will improve the prediction of other AKI related endpoints, these models will require validation using data not available in this dataset.

The simulation studies included here are meant to highlight the complexity of the decision on how to model a partial surrogate for the development of a risk score. This decision is heavily influenced by a mixture model’s ability to choose the correct number of subpopulations for a given problem and resolve subgroup **V** from subgroup **I** by the relationships between covariates and the surrogate outcome. In practice, the only way an analyst can quantify these issues is by fitting a mixture model whenever partial surrogacy is suspected. By inspecting the fitted mixture model, the analyst will then be able to assess the model’s entropy and the clinical significance of the difference between the phenotypes estimated by the mixture model. This provides the analyst with a better understanding of the effect partial surrogacy has on their potential risk score model.

## Conclusions

Recognizing when a clinical marker is acting as a partial surrogate has implications on model selection, predictive ability, and coefficient estimation. Serum creatinine change from baseline, a common marker of kidney injury, displays behavior consistent with a partial surrogate. The use of a mixture model which separates patients into those likely to have a creatinine measure representative of kidney injury and those likely to have an unrelated creatinine measure appears to effectively counter the poor behavior of the biomarker in the cardiac surgery population.

## Additional file

Additional file 1: R Code. (R 6 kb)

## Abbreviations

AKI: Acute kidney injury
eGFR: Glomerular filtration rate estimated by Chronic Kidney Disease Epidemiology Collaboration equation
